# Cell adhesion molecule IGPR-1 activates AMPK connecting cell adhesion to autophagy

**DOI:** 10.1074/jbc.RA120.014790

**Published:** 2021-01-13

**Authors:** Razie Amraei, Tooba Alwani, Rachel Xi-Yeen Ho, Zahra Aryan, Shawn Wang, Nader Rahimi

**Affiliations:** Department of Pathology, School of Medicine, Boston University Medical Campus, Boston, Massachusetts, USA

**Keywords:** autophagy, IGPR-1, IKKβ, AMP-activated kinase (AMPK), post-translational modification (PTM), cell–cell interaction, serine/threonine protein kinase, cell surface receptor, immunoglobulin-like domain, cell adhesion molecule, nutrient deprivation, serine phosphorylation of IGPR-1

## Abstract

Autophagy plays critical roles in the maintenance of endothelial cells in response to cellular stress caused by blood flow. There is growing evidence that both cell adhesion and cell detachment can modulate autophagy, but the mechanisms responsible for this regulation remain unclear. Immunoglobulin and proline-rich receptor-1 (IGPR-1) is a cell adhesion molecule that regulates angiogenesis and endothelial barrier function. In this study, using various biochemical and cellular assays, we demonstrate that IGPR-1 is activated by autophagy-inducing stimuli, such as amino acid starvation, nutrient deprivation, rapamycin, and lipopolysaccharide. Manipulating the IκB kinase β activity coupled with *in vivo* and *in vitro* kinase assays demonstrated that IκB kinase β is a key serine/threonine kinase activated by autophagy stimuli and that it catalyzes phosphorylation of IGPR-1 at Ser^220^. The subsequent activation of IGPR-1, in turn, stimulates phosphorylation of AMP-activated protein kinase, which leads to phosphorylation of the major pro-autophagy proteins ULK1 and Beclin-1 (BECN1), increased LC3-II levels, and accumulation of LC3 punctum. Thus, our data demonstrate that IGPR-1 is activated by autophagy-inducing stimuli and in response regulates autophagy, connecting cell adhesion to autophagy. These findings may have important significance for autophagy-driven pathologies such cardiovascular diseases and cancer and suggest that IGPR-1 may serve as a promising therapeutic target.

Autophagy (also called macroautophagy), the lysosomal degradation of cytoplasmic organelles or cytosolic components, is an evolutionarily conserved cytoprotective mechanism that is induced in response to cellular stress, such as nutrient withdrawal, loss of cell adhesion, and flow shear stress, or by therapeutic genotoxic agents and others (1, 2, 3, 4).

Upon induction of autophagy, unc-51–like kinase 1 (ULK1 also known as ATG1) associates with autophagy-related protein 13 (ATG13) and focal adhesion kinase family interacting protein of 200 kDa (FIP200) to form the ULK1 complex. ULK1 interaction with ATG13 and FIP200 is critical for ULK1 kinase activity and stability (5). The ULK1 complex translocates to autophagy initiation sites and recruits the class III phosphatidylinositol 3-kinase, vacuolar protein sorting 34 (VPS34) complex consisting of BECLIN-1 (the mammalian orthologue of the yeast autophagy protein Apg6/Vps30 (6) and multiple other ATGs leading to the phagophore formation (7). The serine/threonine protein kinase mTOR complex 1 (mTORC1) is a key regulator of autophagy in response to nutrient availability. In the presence of amino acids, mTORC1 is activated and suppresses autophagy through phosphorylation of ULK1 and ATG13. However, upon nutrient deprivation, mTORC1 activity is inhibited, leading to the activation of ULK1 that induces the autophagy program (8, 9). Suppression of mTORC1 activity by AMP-activated protein kinase (AMPK) is central to the regulation of autophagy. AMPK inactivates mTORC1 through phosphorylation of RAPTOR, a key protein present within the mTORC1 complex, and more importantly directly phosphorylates ULK1 at multiple serine residues and activates it (10, 11).

Commonly known autophagy-inducing conditions or agents such as nutrient withdrawal including amino acid and serum starvation, immunosuppressant rapamycin/Sirolimus, and LPS all activate several key kinases such as the IκB kinase (IKK) complex (1). The IKK complex is composed of at least three proteins, including two catalytic subunits (IKKα and IKKβ) and the scaffold protein NF-κB essential modulator (NEMO; also called IKKγ) (12). In addition to its pivotal role in mediating phosphorylation of IκB (12), IKKβ can regulate autophagy in IκB-independent manner (13, 14) by mechanisms that are not fully understood.

IGPR-1 was identified as a novel cell adhesion molecule expressed in various human cell types, including endothelial and epithelial cells, and it mediates cell–cell adhesion (15). IGPR-1 regulates angiogenesis and endothelial barrier function (15, 16), decreases sensitivity of tumor cells to the genotoxic agent doxorubicin, and supports tumor cell survival in response to anoikis (17). IGPR-1 is localized to adherens junctions and is activated through transhomophilic dimerization (16). Additionally, IGPR-1 responds to various cellular stresses, because its phosphorylation (*i.e.* Ser^220^) is significantly increased by flow shear stress (18) and exposure to doxorubicin (17, 18). Curiously, both shear stress (19) and doxorubicin (20, 21) are well-known potent inducers of autophagy, raising a possibility for the involvement of IGPR-1 in autophagy. In this study, we demonstrate that upon the induction of autophagy, IGPR-1 is phosphorylated at Ser^220^ via a mechanism that involves activation of IKKβ. IKKβ-dependent phosphorylation of IGPR-1 stimulates phosphorylation of AMPK, leading to activation of BECN1 and ULK1, connecting cell adhesion and energy sensing to autophagy.

## Results

### IGPR-1 is activated by autophagy

**Figure 1:**
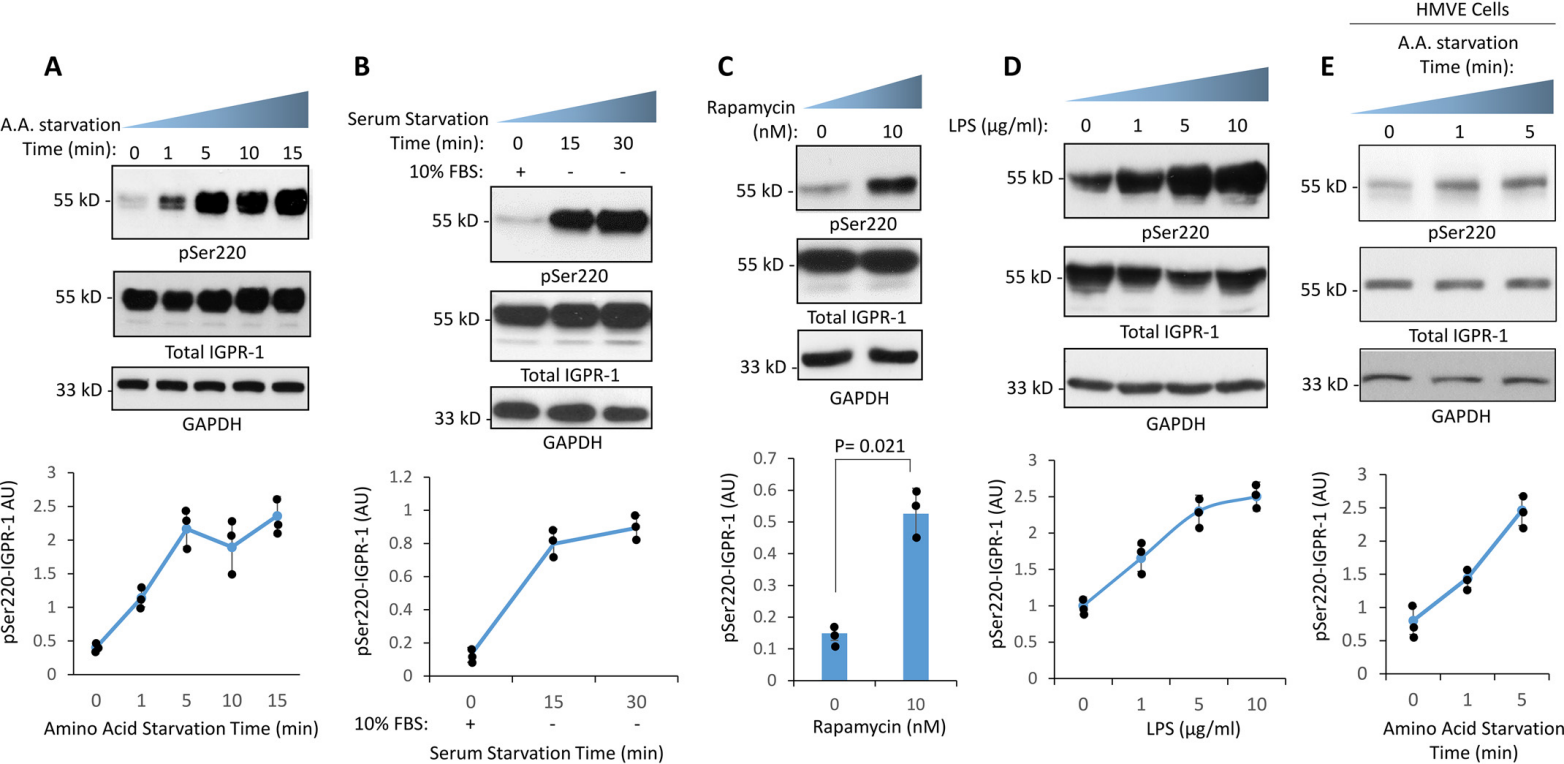
**IGPR-1 is activated by autophagy stimuli.***A*, IGPR-1/HEK-293 cells were kept in 10% FBS or in amino acid–free medium for indicated times. The whole cell lysates were prepared and subjected to Western blotting analysis and blotted with anti-phosphoserine 220 IGPR-1 antibody (pSer^220^), anti–IGPR-1 antibody, and anti-GAPDH antibody for protein loading control. The graph is representative of three independent experiments. Individual data sets are shown (•). *p* < 0.05. *B*, IGPR-1/HEK-293 cells were seeded for 48 h in 10% FBS followed by serum starvation for 15 and 30 min by replacing the 10% DMEM with serum-free DMEM or cells left in 10% FBS DMEM as a control (0). The cells were lysed, and the whole cell lysates were blotted with the same antibodies as *A*. The graph is representative of three independent experiments. Individual data sets are shown (•). *p* < 0.05. *C*, IGPR-1/HEK-293 cells were treated with vehicle (0) or with rapamycin (10 nm) for 1 h. The whole cell lysates were prepared and subjected to Western blotting analysis using the same antibodies as in *A*. The graph is representative of three independent experiments. Individual data sets are shown (•). *p* = 0.021. *D*, IGPR-1/HEK-293 cells were treated with different concentrations of LPS for 1 h as indicated. The whole cell lysates were prepared and subjected to Western blotting analysis and blotted with using the same antibodies as in *A*. The graph is representative of three independent experiments. Individual data sets are shown (•). *p* < 0.05. *E*, HMVECs were kept in 10% FBS or in amino acid–free medium for indicated times. The whole cell lysates were prepared, subjected to Western blotting analysis, and blotted using the same antibodies as in *A*. Individual data sets are shown (•). *P* < 0.05.

Homophilic transdimerization of IGPR-1 regulates its phosphorylation at Ser^220^ (16). Additionally, genotoxic agents such as doxorubicin (17) and flow shear stress (18) also simulate phosphorylation of IGPR-1at Ser^220^. Because both shear stress and genotoxic agents are well-known for their roles in autophagy, we asked whether IGPR-1 is activated in response to autophagy. We used human embryonic kidney epithelial-293 (HEK-293) cells ectopically expressing IGPR-1 as a model system to study the role of IGPR-1 in autophagy, because these cells do not express IGPR-1 endogenously at a detectable level (16). To this end, we first, tested whether amino acid starvation, the best-known inducer of autophagy, can stimulate phosphorylation of IGPR-1. The cells were lysed, and whole cell lysates were subjected to Western blotting analysis followed by immunoblotting with pSer^220^ and total IGPR-1 antibodies. Phosphorylation of IGPR-1 at Ser^220^ was significantly increased by brief amino acid starvation of HEK-293 cells. The increase in phosphorylation of Ser^220^ peaked after 1 min with amino acid starvation and remained highly phosphorylated until 15 min (Fig. 1*A*).

Furthermore, in an additional set of experiments we subjected HEK-293 cells expressing IGPR-1 to various other autophagy-inducing conditions or factors such as serum-starvation, rapamycin, and LPS treatments and measured phosphorylation of Ser^220^. Both rapamycin and LPS are known to induce autophagy (1, 22, 23). Phosphorylation of IGPR-1 at Ser^220^ was significantly increased by brief serum starvation of HEK-293 cells (Fig. 1*B*). Furthermore, both rapamycin and LPS treatments of HEK-293 cells stimulated phosphorylation of IGPR-1 at Ser^220^ (Fig. 1, *C* and *D*). To demonstrate whether IGPR-1 is phosphorylated by autophagy in biologically relevant human endothelial cells in which IGPR-1 is expressed endogenously, we subjected primary human microvascular endothelial cells (HMVECs) to amino acid starvation. The result showed that IGPR-1 is phosphorylated at Ser^220^ in HMVECs (Fig. 1*E*). Taken together, the data demonstrate that IGPR-1 is activated by autophagy in HEK-293 cells and human primary endothelial cells.

### IKKβ is activated by autophagy and phosphorylates IGPR-1

**Figure 2:**
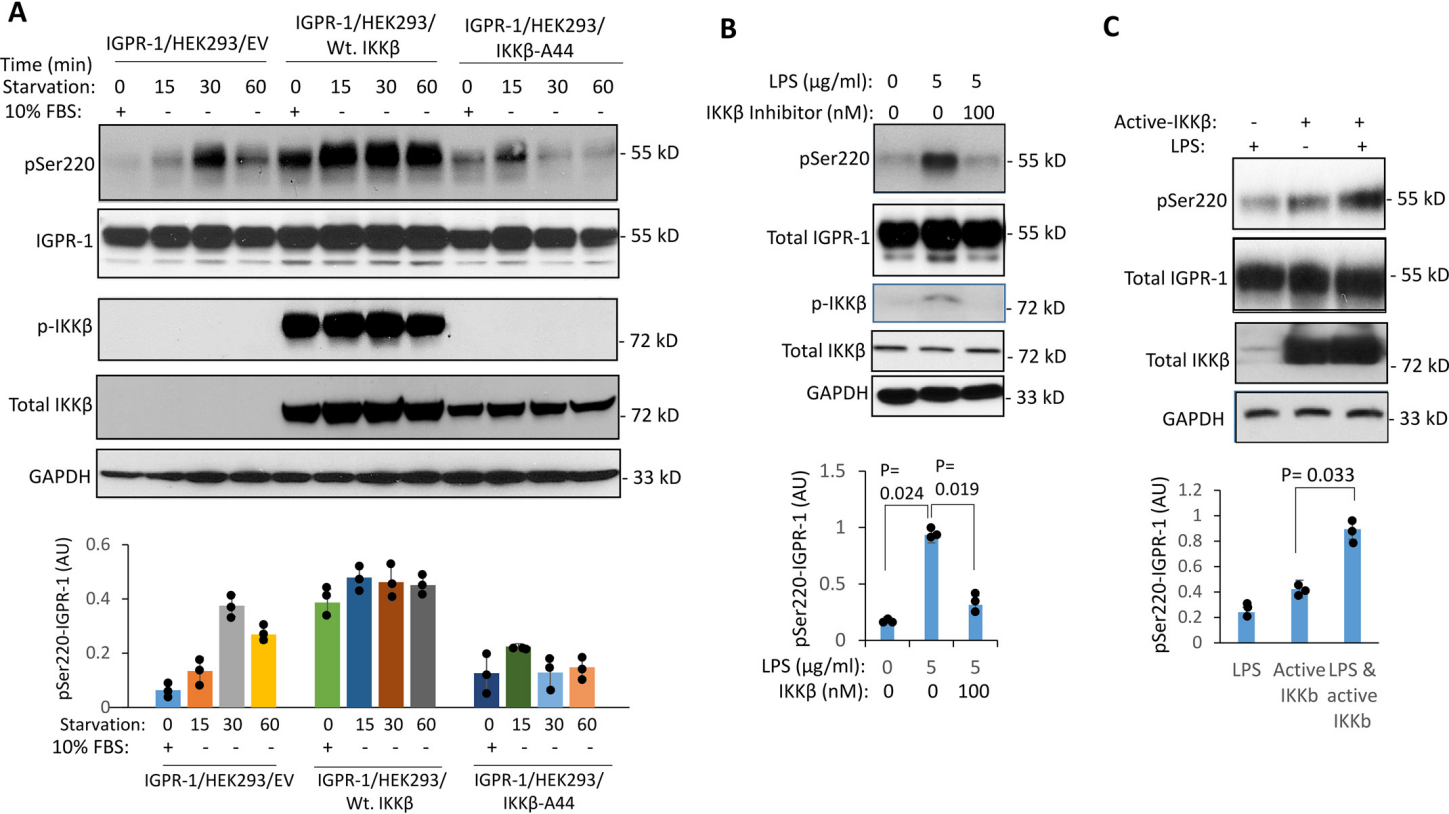
**IKKβ mediates serum starvation–dependent and LPS-induced activation of IGPR-1.***A*, IGPR-1/HEK293 cells were transfected with an EV, WT IKKβ (*Wt.IKK*β), or kinase-dead IKKβ (*IKK*β*-A44*). After 48 h of transfection, the cells were either left in 10% FBS DMEM or serum-starved for 15 or 30 min. The cells were lysed, and the whole cell lysates were immunoblotted for pSer^220^, total IGPR-1, phospho-IKKβ, or total IKKβ or blotted for GAPDH for protein loading control. The graph is representative of three independent experiments. Individual data sets are shown (•). *p* < 0.05. *B*, IGPR-1/HEK-293 cells were treated with LPS (5 µg/ml) alone or cotreated with LPS and IKK inhibitor III/BMS-345541 (100 ηm) for 60 min. The cells were lysed, and the whole cell lysates were subjected to Western blotting analysis and probed for pSer^220^, total IGPR-1, phospho-IKKβ, total IKKβ, and GAPDH for protein loading control. The graph is representative of three independent experiments. Individual data sets are shown (•). *C*, IGPR-1/HEK-293 cells were transfected with EV (−) or constitutively active IKKβ (IKKβ-S177E/S181E). After 48 h the cells were either left untreated (−) or treated with LPS (5 µg/ml) for 60 min (+). The whole cell lysates were subjected to Western blotting analysis and probed for pSer^220^, total IGPR-1, total IKKβ, and GAPDH for protein loading control. The graph is representative of three independent experiments. Individual data sets are shown (•). *p* = 0.033.

Activation of serine/threonine kinases represents a salient mechanistic feature of autophagy. Particularly, activation of IKKβ by nutrient deprivation (24), LPS (25, 26), and rapamycin (27) is known to play a central role in autophagy. Therefore, we asked whether activation of IKKβ by serum starvation, LPS, or rapamycin can mediate phosphorylation of IGPR-1 at Ser^220^. To this end, we overexpressed WT IKKβ or kinase inactive IKKβ (IKKβ-A44) in IGPR-1/HEK-293 cells. After 48 h of transfection, the cells were either kept in 10% FBS or serum-starved for 15, 30, or 60 min. The cells were lysed, and whole cell lysates were immunoblotted for pSer^220^, total IGPR-1, and phospho-IKKβ. Expression of WT IKKβ in IGPR-1/HEK-293 cells resulted in a robust phosphorylation of IGPR-1 in the presence of 10% FBS, and this was further increased in response to serum starvation (Fig. 2*A*). In contrast, overexpression of kinase inactive IKKβ-A44 in IGPR-1/HEK-293 cells markedly reduced phosphorylation of IGPR-1 at Ser^220^ (Fig. 2*A*), indicating that IKKβ kinase activity is required for the serum starvation–dependent phosphorylation of IGPR-1 at Ser^220^. Similarly, LPS-induced phosphorylation of Ser^220^ on IGPR-1 was inhibited by a selective IKKβ inhibitor, IKK inhibitor III/BMS-345541 (28) (Fig. 2*B*). Furthermore, LPS stimulated phosphorylation of IKKβ and IKK inhibitor blocked its phosphorylation (Fig. 2*B*). Conversely, overexpression of a constitutively active IKKβ (IKKβ-S177E/S181E) in IGPR-1/HEK293 cells augmented LPS-induced phosphorylation of Ser^220^ (Fig. 2*C*), indicating that IKKβ activity also is required for LPS-induced phosphorylation of IGPR-1 at Ser^220^.

### IGPR-1 is a substrate for IKKβ and is phosphorylated by IKKβ in vitro and in vivo

**Figure 3:**
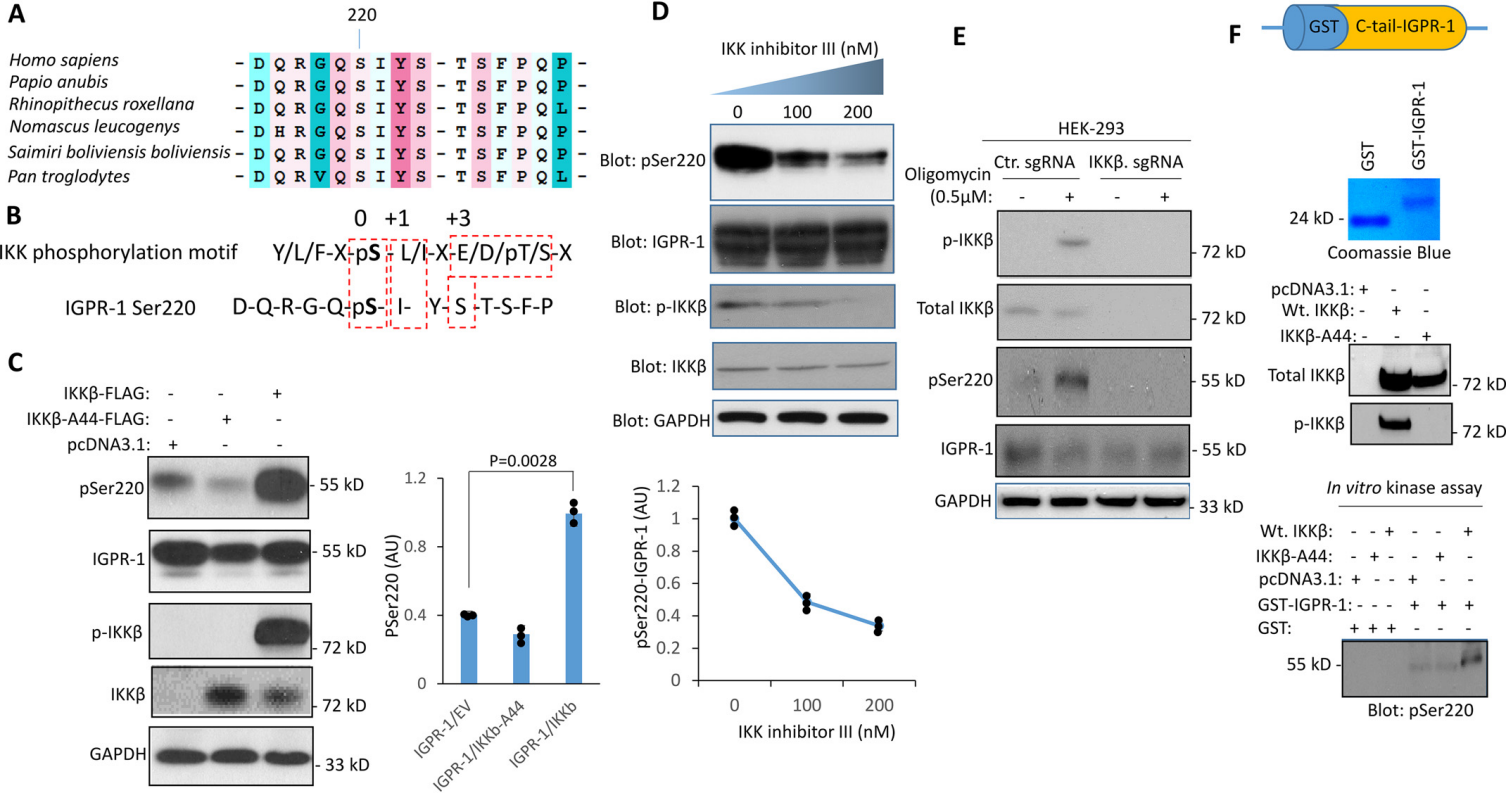
**IGPR-1 is a substrate of and phosphorylated by IKKβ.***A*, Ser^220^ and the surrounding amino acids on IGPR-1 in human and nonhuman primates is conserved. *B*, IKKβ phosphorylation motif and IGPR-1 Ser^220^ phosphorylation site. *C*, IGPR-1/HEK-293 cells were transfected with WT IKKβ or kinase inactive IKKβ (*IKK*β*-A44*). After 48 h the cells were lysed and immunoblotted for pSer^220^, total IGPR-1, phospho-IKKβ, total IKKβ, and GAPDH. The graph is representative of three independent experiments. Individual data sets are shown (•). *p* = 0.0028. *D*, IGPR-1/HEK-293 cells were treated with IKK inhibitor III/BMS-345541 (100 ηm) for 60 min. The cells were lysed and immunoblotted for pSer^220^, total IGPR-1, phospho-IKKβ, and total IKKβ. The graph is representative of three independent experiments. Individual data sets are shown (•). *p* < 0.05. *E*, Ctr.sgRNA/IGPR-1/HEK-293 cells and IKKβ-sgRNA/IGPR-1/HEK-293 cells were stimulated with IKKβ activator, Oligomycin (0.5 μm). The cells were lysed, subjected to Western blotting analysis, and immunoblotted for the indicated proteins. *F*, *in vitro* kinase assay was performed by incubation of purified GST-cytoplasmic domain of IGPR-1 and purified WT IKKβ or kinase inactive IKKβ-A44 in the presence of ATP. *In vitro* kinase reaction was stopped after 30 min by heating the samples at 90 °C for 5 min. The samples were subjected to Western blotting analysis, and phosphorylation of Ser^220^ on GST–IGPR-1 was detected by immunoblotting with pSer^220^ antibody.

Ser^220^ and the surrounding amino acids in IGPR-1 are strongly conserved both in human and nonhuman primates (Fig. 3*A*), suggesting an evolutionarily conserved mechanism for the phosphorylation of Ser^220^. IKKβ phosphorylates peptides with aromatic residues at the −2 position, hydrophobic residues at the +1 position, and acidic residues at the +3 position (29), suggesting that IKKβ is a likely candidate kinase involved in the phosphorylation of IGPR-1 at Ser^220^ (Fig. 3*B*). Therefore, we asked whether IKKβ can phosphorylate IGPR-1 at Ser^220^ independent of the autophagy-inducing factors like serum starvation or LPS and rapamycin. We overexpressed WT IKKβ or kinase inactive IKKβ-A44 in IGPR-1/HEK-293, and 48 h after transfection, the cells were lysed, and phosphorylation of IGPR-1 was determined by Western blotting analysis. Overexpression of WT IKKβ increased phosphorylation of IGPR-1, whereas kinase inactive IKKβ-A44 inhibited phosphorylation of IGPR-1 at Ser^220^ (Fig. 3*C*). Similarly, IKKβ inhibitor inhibited both phosphorylation of IGPR-1 and IKKβ (Fig. 3*D*).

In an additional approach, we knocked out IKKβ via CRISPR-Cas9 system and examined the effect of loss of IKKβ in IGPR-1 phosphorylation. To this end, cells were either treated with a control vehicle or AMPK activator, oligomycin and cells were lysed, and phosphorylation of IGPR-1 at Ser^220^ was determined. Stimulation of IGPR-1/Ctr.sgRNA/HEK-293 cells with oligomycin stimulated AMPK activation and phosphorylation of IGPR-1 at Ser^220^ (Fig. 3*E*). However, in IGPR-1/IKKβ.sgRNA/HEK-293 cells, phosphorylation of Ser^220^ was not detected, and treatment with oligomycin also did not stimulate phosphorylation of IGPR-1 at Ser^220^ (Fig. 3*E*). IKKβ.sgRNA-mediated knockout of IKKβ is shown (Fig. 3*E*).

We next asked whether IKKβ can directly phosphorylates IGPR-1 at Ser^220^. To examine the direct involvement of IKKβ in catalyzing the phosphorylation of IGPR-1, we carried out an *in vitro* kinase assay using a purified recombinant GST–IGPR-1 protein that only encompasses the cytoplasmic domain of IGPR-1 and demonstrated that IKKβ phosphorylates IGPR-1 at Ser^220^ (Fig. 3*F*). Taken together, our data identify IGPR-1 as a novel substrate of IKKβ. Furthermore, we carried additional experiments to demonstrate the selectively of IGPR-1 phosphorylation at Ser^220^ by IKKβ. To this end, we immunoprecipitated IGPR-1 proteins from HEK-293 cells expressing either WT IGPR-1, A220–IGPR-1, or D220–IGPR-1. The immunoprecipitated proteins were subjected to calf intestinal alkaline phosphatase treatment, which removes phosphorylation. The removal of phosphorylation on IGPR-1 was confirmed by Western blotting analysis using pSer^220^ specific antibody (Fig. S1A). The dephosphorylated proteins were subjected to an *in vitro* kinase assay using a recombinant IKKβ. The result showed that IKKβ selectively phosphorylates IGPR-1 at Ser^220^ (Fig. S1B).

### IGPR-1 activates AMPK and stimulates phosphorylation of BECN1 and ULK1

**Figure 4:**
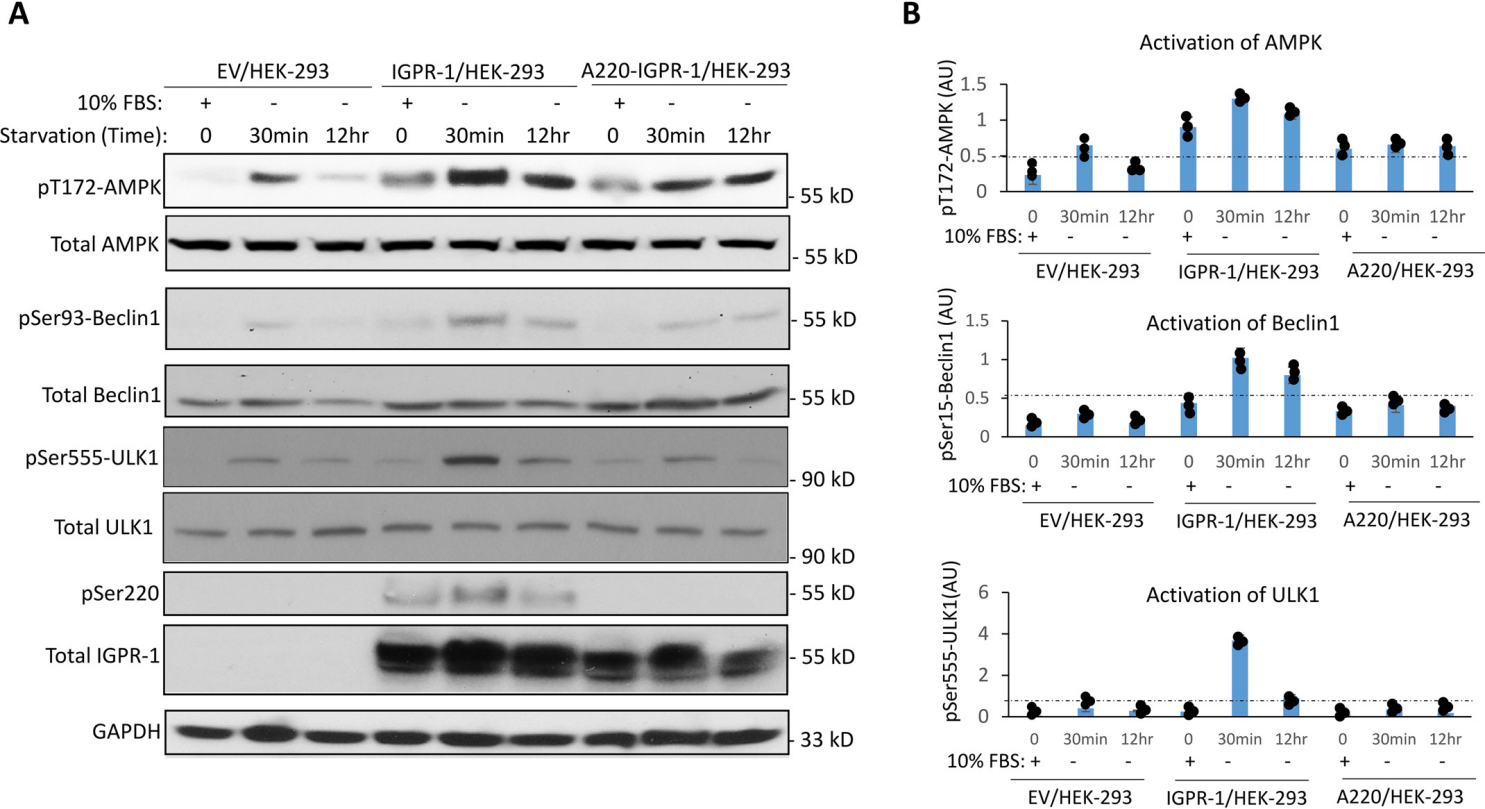
**IGPR-1 stimulates phosphorylation of AMPK and mediates phosphorylation of ULK1.***A*, HEK-293 cells expressing EV, IGPR-1, or A220–IGPR-1 were either kept in 10% FBS DMEM or starved for 30 min or 12 h. The cells were lysed and immunoblotted for pT172-AMPK, pSer555-ULK1, pSer15-Beclin1, pSer^220^–IGPR-1, total IGPR-1, and GAPDH. *B*, graphs showing activation of AMPK, Beclin1, and ULK1 are representative of three independent experiments. Individual data sets are shown (•). *p* < 0.05.

We sought to determine whether IGPR-1 could activate AMPK, a widely considered master regulator of autophagy (10). HEK-293 cells expressing an empty vector (EV), IGPR-1, or A220–IGPR-1 were either maintained in 10% FBS or serum-starved for 30 min or 12 h. The pThr^172^-AMPK immunoblot of whole cell lysates showed that expression of IGPR-1 in HEK-293 cells bypassed the requirement for serum starvation–dependent activation of AMPK because AMPK was strongly phosphorylated at Thr^172^ in IGPR-1/HEK-293 cells in 10% FBS compared with EV/HEK-293 cells (Fig. 4*A*). Phosphorylation of AMPK was further augmented in IGPR-1/HEK-293 cells, particularly at 30 min, compared with control EV/HEK-293 cells (Fig. 4*A*). Interestingly, A220–IGPR-1/HEK-293 cells showed also an increase in phosphorylation of AMPK, notwithstanding significantly less than the WT IGPR-1, but more than the control EV/HEK-293 cells (Fig. 4*A*). Phosphorylation of AMPKα at Thr^172^ in the activation loop is required for AMPK activation (30, 31), indicating that expression of IGPR-1 in HEK-293 cells activated AMPK.

We next asked whether IGPR-1 induced AMPK activation also stimulates phosphorylation of BECN1 and ULK1, which are the best-known substrates of AMPK and play central roles in autophagy (32). Similar to AMPK activation, phosphorylation of BECN1 at Ser^93^ was significantly increased in IGPR-1/HEK-293 cells compared with EV/HEK-293 or A220–IGPR-1 cells (Fig. 4*A*), indicating that IGPR-1–induced activation of AMPK in HEK-293 cells also induced phosphorylation of BECN1 (Fig. 4*A*) as previously reported (33). Furthermore, phosphorylation of ULK1 at Ser^555^ was also increased in IGPR-1/HEK-293 cells compared with control EV/HEK-293 cells. Interestingly, ULK1 phosphorylation was significantly reduced in A220–IGPR-1 cells, indicating that phosphorylation of Ser^220^, in part, is required for phosphorylation of ULK-1 (Fig. 4*A*). The data indicate that phosphorylation of Ser^220^ on IGPR-1 plays an important role in the phosphorylation of AMPK, BECN1, and ULK1.

### IGPR-1 induces autophagy in HEK-293 cells

**Figure 5:**
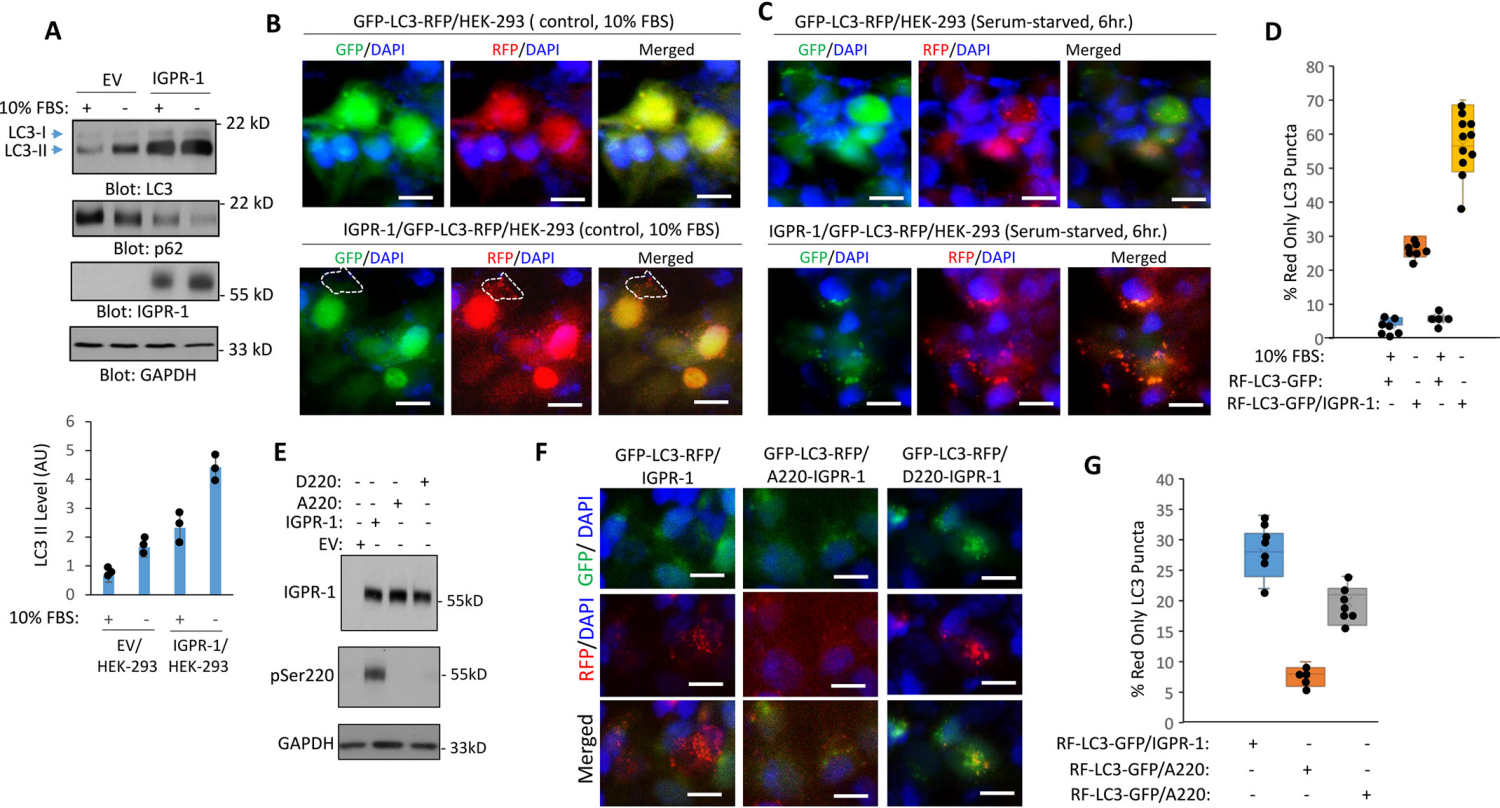
**IGPR-1 mediates serum starvation–induced autophagy.***A*, HEK-293 cells expressing EV, IGPR-1 were either kept in 10% FBS or serum-starved for 12 h. The cells were lysed and immunoblotted for LC3 and p26, total IGPR-1, and GAPDH. The graph is representative of three experiments. *B* and *C*, HEK-293 cells expressing GFP-LC3-RFP/HEK-293 alone or coexpressing GFP-LC3-RFP and IGPR-1 either were kept in 10% FBS DMEM (*B*) or in serum-free DMEM for 6 h (*C*). The cells were fixed and stained with 4′,6-diamino-2-phenylindole (*DAPI*, nucleus) and viewed under a fluorescence microscope, and pictures were taken. *Scale bar*, 10 μm. *D*, the graph is representative of three independent experiments. ImageJ was used to quantify images (*n* = 6–10 images for group). Individual data sets are shown (•). *p* < 0.05. *E*, expression and phosphorylation of IGPR-1, A220–IGPR-1, or D220–IGPR-1 in HEK-293 cells. *F*, HEK-293 cells coexpressing GFP-LC3-RFP with IGPR-1, A220–IGPR-1, or D220–IGPR-1 were kept in serum-free DMEM for 6 h. The cells were fixed and stained with as in *A* and viewed under a fluorescence microscope, and representative pictures were taken. *Scale bar*, 10 μm. *G*, ImageJ was used to quantify images (*n* = 6–10 images for group). Individual data sets are shown (•). *p* < 0.05.

We next examined the role of IGPR-1 in the autophagosome formation by measuring expression of LC3-phosphatidylethanolamine conjugate (LC3-II) and p62 endogenously expressed in HEK-293 cells, which are required for autophagosome development during autophagy (34), by Western blotting analysis. HEK-293 cells expressing EV or IGPR-1 were either kept at 10% FBS or serum-starved for overnight. The cells were lysed, and expression of LC3 and p62 levels was determined by immunoblotting with LC3 and p62 antibodies. Although expression of LC3II was increased in response to serum starvation in EV/HEK-293 cells, expression of LC3II was significantly higher both in 10% FBS and in serum-starved conditions in IGPR-1/HEK-293 cells (Fig. 5*A*), indicating that IGPR-1 through increased in expression of LC3II regulates autophagosome formation. Additionally, as expected, p62 level was markedly decreased in response to serum starvation (Fig. 5*A*).

To further elucidate the role of IGPR-1 in induction of autophagy, we established autophagic flux reporter cell lines by creating GFP-LC3 (microtubule-associated protein 1 light chain 3β)–RFP/HEK-293 and IGPR-1/GFP-LC3-RFP/HEK-293 cell lines via a retroviral expression system as previously reported (35). During autophagy, GFP-LC3-RFP labeled autophagosomes fuse with lysosomes. Although the GFP signals are quenched because of the acidic environment (GFP is acid-sensitive) in the autolysosomes, the RFP signals remain stable because RFP is acid-stable, and hence an increase in the number of RFP-LC3 (red only) puncta is considered a reflection of autophagic flux (36). In the presence of 10% FBS, only a few RFP-LC3 positive puncta were observed in GFP-LC3-RFP/HEK-293 cells (Fig. 5, *B* and *D*). However, we observed a significantly higher baseline of RFP-LC3–positive puncta, but not GFP-LC3–positive puncta, in IGPR-1/GFP-LC3-RFP/HEK-293 cells (Fig. 5, *C* and *D*). Moreover, when IGPR-1/GFP-LC3-RFP/HEK-293 cells were induced to undergo autophagy by serum starvation, they displayed a substantial increase in RFP-LC3–positive puncta (Fig. 5, *C* and *D*), suggesting that IGPR-1 regulates the formation of autophagosomes.

Next, we asked whether Ser^220^ mutant IGPR-1 (A220–IGPR-1) can induce RFP-LC3 puncta formation in HEK-293 cells. Expression of IGPR-1, A220–IGPR-1, or D220–IGPR-1 in HEK-293 cells (Fig. 5*E*). The result showed that the ability of A220–IGPR-1 to induce RFP-LC3–positive puncta was significantly reduced (Fig. 5, *E* and *G*). However, the phosphomimetic mutation, D220–IGPR-1, largely maintained IGPR-1–mediated autophagic flux (Fig. 5, *F* and *G*). Altogether, the data demonstrate that IGPR-1 is activated by and regulates autophagy program.

## Discussion

Previous studies have shown that the activation of IKKβ regulates autophagy through mechanisms that involve expression of pro-autophagic genes via the NF-κB–independent pathway and phosphorylation of the p85 subunit of PI3K, which leads to inhibition of mTOR (13, 24). We revealed the existence of a previously unidentified pathway in autophagy that involves IKKβ-dependent activation of IGPR-1. We provide a mechanistic link between activation of IKKβ and phosphorylation of IGPR-1 at Ser^220^. IKKβ is activated by autophagy, leading to phosphorylation of IGPR-1 at Ser^220^ both *in vivo* and *in vitro*. Mechanistically, IKKβ-mediated phosphorylation of IGPR-1 at Ser^220^ leads to activation of AMPK, which plays a central role in autophagy. Activation of IKKβ plays an essential role in autophagy because both the loss of function of IKKβ in mice and cell culture blocked autophagy (13). Likewise, IKKβ null cells are deficient in their ability to undergo autophagy in response to cellular starvation (14), which further underscores the critical role of IKKβ in autophagy.

AMPK is believed to exert its effect in autophagy by multiple mechanisms including, inactivating mTORC1 through phosphorylation of RAPTOR, a key protein present within the mTORC1, phosphorylating ULK1 at multiple serine residues, including Ser^555^, that lead to its activation (10, 11) and more importantly phosphorylating BECN1 at Ser^93^ and Ser^96^ (33). IGPR-1–mediated activation of AMPK in HEK-293 cells increased phosphorylation of ULK1 at Ser555 and BECN1 at Ser93. Activation of AMPK and phosphorylation of BECN1 requires phosphorylation of IGPR-1 at Ser^220^. Activation of ULK1 and phosphorylation of BECN1 both play central roles in autophagy. ULK1 phosphorylation enables ULK1 to form a complex with ATG13 and FIP200 (focal adhesion kinase family interacting protein of 200 kDa) that leads to its translocation to autophagy initiation sites and subsequent recruitment of the class III phosphatidylinositol 3-kinase, VPS34 (vacuolar protein sorting 34) complex consisting of BECN1 and multiple other autophagy-related proteins leading to the phagophore formation (6). Phosphorylation of BECN1 plays a key role in the initial steps in the assembly of autophagosomes from preautophagic structures, which is the recruitment and activation of VPS34 complex (32).

IGPR-1 is a cell adhesion molecule that mediates cell–cell adhesion, and its activation regulates cell morphology and actin stress fiber alignment (15, 16). The finding that IGPR-1 is activated by and regulates autophagy by stimulating activation of AMPK not only suggests a significant role for IGPR-1 in autophagy but also links cell–cell adhesion to energy sensing and autophagy. Recent studies illuminated the key roles of autophagy in endothelial cells in response to various metabolic, blood flow–induced stresses and angiogenesis (37), the same cellular events are also regulated by IGPR-1 (15, 16, 18).

Additionally, cellular stress induced by flow shear stress (19, 38) and exposure of cells to the chemotherapeutic agent doxorubicin (20, 21) are linked to induction of autophagy, the conditions in which IGPR-1 also is activated (17, 18). Moreover, autophagy is associated with therapeutic resistance to chemotherapeutic agents (*e.g.* cisplatin, doxorubicin, temozolomide, and etoposide), metabolic stresses, and small molecule inhibitors, suggesting a pro-tumor function for autophagy (39, 40). Curiously, IGPR-1 is strongly phosphorylated by doxorubicin and regulates sensitivity of tumor cells to doxorubicin (17), indicating that IGPR-1 through induction of autophagy program could contribute to the development of resistance in cancer cells.

**Figure 6:**
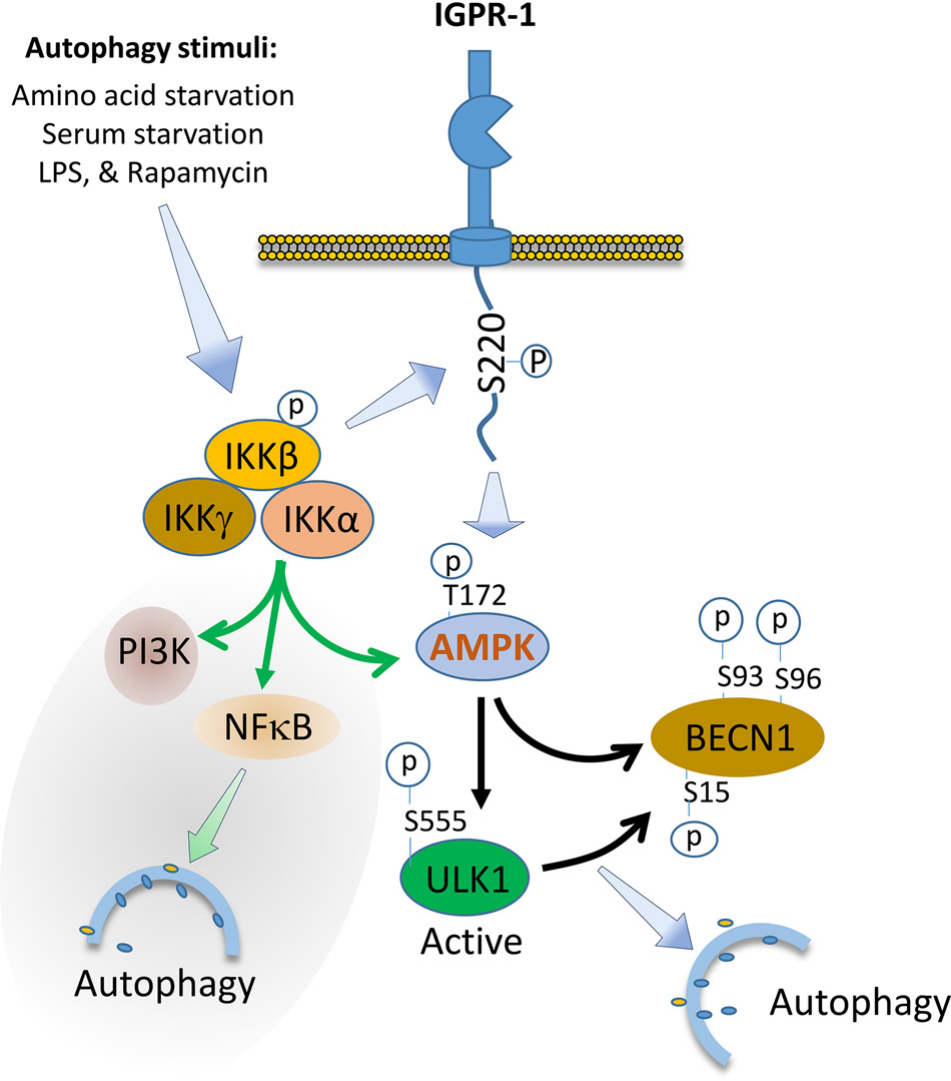
**Proposed role of IGPR-1 in autophagy.** Upon activation by autophagy stimuli, IGPR-1 is phosphorylated at Ser^220^ via IKKβ. IGPR-1 acts as a pro-autophagy signaling receptor leading to activation of AMPK. AMPK catalyzes phosphorylation of BECN1 and ULK1, key proteins involved in autophagy. IGPR-1 is a dimeric protein and undergoes homophilic transdimerization in a cell density–dependent manner (17) (not shown). Activation of IKKβ was previously thought to regulate autophagy by activation of AMPK, PI3K, and induction of NF-κB.

Taken together, the data presented here suggest a significant role for IGPR-1 in autophagy and autophagy-associated diseases such as cancer and cardiovascular diseases. We propose IGPR-1 as a pro-autophagy cell adhesion molecule that upon activation stimulates AMPK activation, leading to phosphorylation of BECN1 and ULK1, key proteins involved in autophagy (Fig. 6), linking cell adhesion to autophagy, a finding that has important significance for autophagy-driven pathologies such cardiovascular diseases and cancer.

## Materials and methods

### Antibodies, plasmids, sgRNAs, and chemicals

Anti–IGPR-1 and anti-pSer^220^ antibodies are homemade rabbit polyclonal antibodies previously described (15, 16). Phospho-AMPK (Thr^172^), total AMPK, phospho-ULK1 (Ser^555^), total ULK1, phosph-Beclin-1 (Ser^93^), total Beclin-1, phospho-IKK (Ser^176/180^), total IKKβ, and LC3A/B, GAPDH antibodies all were purchased from Cell Signaling Technologies (Danvers, MA, USA). The following plasmids were all purchased from Addgene (Watertown, MA, USA): pcDNA3.FLAG-ULK1 (catalog no. 27636), pMRX-IP-GFP-LC3-RFP (catalog no. 84573), pcDNA-IKKβ-FLAG (catalog no. 23298), pcDNA-IKKβ-A44 (catalog no. 23299), and constitutive active IKKβ (S177E S181E, catalog no. 11105). IGPR-1 constructs including WT IGPR-1 and Ser^220^ mutant, A220–IGPR-1 constructs were cloned into retroviral vector, pQCXIP with C-terminal Myc tag as previously described (16, 17, 18). Retroviruses were produced in 293-GPG cells as described (41). IKK inhibitor III and rapamycin were purchased from Calbiochem, and LPS was purchased from Sigma. Oligomycin was purchased from Cell Signaling Technologies. A set of three human IKKβ sgRNAs (catalog no. GSGH-11938-16EG3551) were purchased from Dharmacon (Chicago, IL, USA).

### Cell culture assays

HEK-293 cells expressing EV, IGPR-1 or A220–IGPR-1 were maintained in DMEM supplemented with 10% fetal bovine serum and penicillin/streptomycin. To measure phosphorylation of IGPR-1 in response to serum starvation, the cells were plated in 60-cm plates with 10% FBS DMEM overnight with approximate 80–90% confluency. The cells were washed twice with PBS, and the cells were starved for 15 and 30 min or as described in the figure legends. The cells were lysed, and the whole cell lysates were mixed with sample buffer (5×) and boiled for 5 min. The whole cell lysates were subjected to Western blotting analysis and immunoblotted with antibody of the interest as described in the figure legends. In some experiments, the cells were treated with a specific chemical inhibitor or transfected with a particular construct as indicated in the figure legends. Human microvascular endothelial cells were purchased from Cell Applications, Inc. (San Diego, CA, USA) and were grown in endothelial cell medium.

### Recombinant GST-fusion protein production

The generation of GST-fusion cytoplasmic domain of IGPR-1 cloned into pGEX-2T vector as previously described (15). The purified GST-fusion IGPR-1 protein was subsequently used to measure the ability of IKKβ to phosphorylate IGPR-1 in an *in vitro* kinase.

### In vitro kinase assay

To detect phosphorylation of IGPR-1 at Ser^220^, the purified recombinant GST–IGPR-1 encompassing the cytoplasmic domain of IGPR-1 was mixed with WT or kinase inactive IKKβ expressed in HEK-293 cells in 1× kinase buffer plus 0.2 mm ATP and incubated at 30 °C for 15 min. The samples were mixed in 2× sample buffer and after boiling at 95 °C for 5 min were resolved on 12% SDS–PAGE followed by Western blotting analysis using anti-pSer^220^ antibody.

### Western blotting analysis

The cells were prepared as described in the figure legends and lysed, and the whole cell lysates were subjected to Western blotting analysis. Normalized whole cell lysates were subjected to Western blotting analysis using IGPR-1 antibody, pSer^220^ antibodies, or an appropriate antibody as indicated in the figure legends. The proteins were visualized using streptavidin–horseradish peroxidase–conjugated secondary antibody via chemiluminescence system. For each blot, the films were exposed multiple times, and the films that showed within the linear range detection of protein bands were selected, scanned, and subsequently used for quantification. Blots from at least three independent experiments were used for quantification purposes, and representative data are shown. ImageJ software, an open source image-processing program, was used to quantify the blots.

### Immunofluorescence microscopy

The cells expressing IGPR-1 or other constructs were seeded (1.5 × 10^6^ cells) onto coverslips and grown overnight in 60-mm plates to 90–100% confluence. The coverslips were mounted in Vectashield mounting medium with 4′,6-diamino-2-phenylindole onto glass microscope slides. The slides were examined using a fluorescence microscope.

### Statistical analyses

The experimental data were subjected to Student's *t* test or one-way analysis of variance analysis where appropriate with representative of at least three independent experiments. *p* < 0.05 was considered significant or as indicated in the figure legends.

## Data availability

The data and reagents are available from the corresponding author upon request.

10.13039/100011541HHS | NIH | NCI | Division of Cancer Epidemiology and Genetics, National Cancer Institute (DCEG) (R21CA191970, R21CA193958) to Nader Rahimi

## References

[1] Kroemer G., Mariño G., Levine B. (2010). Autophagy and the integrated stress response. Mol. Cell.

[2] Vlahakis A., Debnath J. (2017). The interconnections between autophagy and integrin-mediated cell adhesion. J. Mol. Biol.

[3] Kenific C.M., Wittmann T., Debnath J. (2016). Autophagy in adhesion and migration. J. Cell Sci.

[4] Fung C., Lock R., Gao S., Salas E., Debnath J. (2008). Induction of autophagy during extracellular matrix detachment promotes cell survival. Mol. Biol. Cell.

[5] Hurley J.H., Young L.N. (2017). Mechanisms of autophagy initiation. Annu. Rev. Biochem.

[6] Kametaka S., Okano T., Ohsumi M., Ohsumi Y. (1998). Apg14p and Apg6/Vps30p form a protein complex essential for autophagy in the yeast, *Saccharomyces cerevisiae*. J. Biol. Chem.

[7] Huang W.P., Klionsky D.J. (2002). Autophagy in yeast: a review of the molecular machinery. Cell Struct. Funct.

[8] Jung C.H., Jun C.B., Ro S.H., Kim Y.M., Otto N.M., Cao J., Kundu M., Kim D.H. (2009). ULK–Atg13–FIP200 complexes mediate mTOR signaling to the autophagy machinery. Mol. Biol. Cell.

[9] Hosokawa N., Hara T., Kaizuka T., Kishi C., Takamura A., Miura Y., Iemura S., Natsume T., Takehana K., Yamada N., Guan J.L., Oshiro N., Mizushima N. (2009). Nutrient-dependent mTORC1 association with the ULK1–Atg13–FIP200 complex required for autophagy. Mol. Biol. Cell.

[10] Kim J., Kundu M., Viollet B., Guan K.-L. (2011). AMPK and mTOR regulate autophagy through direct phosphorylation of Ulk1. Nat. Cell Biol.

[11] Egan D.F., Shackelford D.B., Mihaylova M.M., Gelino S., Kohnz R.A., Mair W., Vasquez D.S., Joshi A., Gwinn D.M., Taylor R., Asara J.M., Fitzpatrick J., Dillin A., Viollet B., Kundu M. (2011). Phosphorylation of ULK1 (hATG1) by AMP-activated protein kinase connects energy sensing to mitophagy. Science.

[12] Perkins N.D. (2007). Integrating cell-signalling pathways with NF-κB and IKK function. Nat. Rev. Mol. Cell Biol.

[13] Criollo A., Senovilla L., Authier H., Maiuri M.C., Morselli E., Vitale I., Kepp O., Tasdemir E., Galluzzi L., Shen S., Tailler M., Delahaye N., Tesniere A., De Stefano D., Younes A.B. (2010). The IKK complex contributes to the induction of autophagy. EMBO J.

[14] Comb W.C., Cogswell P., Sitcheran R., Baldwin A.S. (2011). IKK-dependent, NF-κB–independent control of autophagic gene expression. Oncogene.

[15] Rahimi N., Rezazadeh K., Mahoney J.E., Hartsough E., Meyer R.D. (2012). Identification of IGPR-1 as a novel adhesion molecule involved in angiogenesis. Mol. Biol. Cell.

[16] Wang Y.H.W., Meyer R.D., Bondzie P.A., Jiang Y., Rahimi I., Rezazadeh K., Mehta M., Laver N.M.V., Costello C.E., Rahimi N. (2016). IGPR-1 is required for endothelial cell–cell adhesion and barrier function. J. Mol. Biol.

[17] Woolf N., Pearson B.E., Bondzie P.A., Meyer R.D., Lavaei M., Belkina A.C., Chitalia V., Rahimi N. (2017). Targeting tumor multicellular aggregation through IGPR-1 inhibits colon cancer growth and improves chemotherapy. Oncogenesis.

[18] Ho R.X., Tahboub R., Amraei R., Meyer R.D., Varongchayakul N., Grinstaff M., Rahimi N. (2019). The cell adhesion molecule IGPR-1 is activated by, and regulates responses of endothelial cells to shear stress. J. Biol. Chem.

[19] Liu J., Bi X., Chen T., Zhang Q., Wang S.X., Chiu J.J., Liu G.S., Zhang Y., Bu P., Jiang F. (2015). Shear stress regulates endothelial cell autophagy via redox regulation and Sirt1 expression. Cell Death Dis.

[20] Chen H., Zhao C., He R., Zhou M., Liu Y., Guo X., Wang M., Zhu F., Qin R., Li X. (2019). Danthron suppresses autophagy and sensitizes pancreatic cancer cells to doxorubicin. Toxicol In Vitro.

[21] Sui X., Chen R., Wang Z., Huang Z., Kong N., Zhang M., Han W., Lou F., Yang J., Zhang Q., Wang X., He C., Pan H. (2013). Autophagy and chemotherapy resistance: a promising therapeutic target for cancer treatment. Cell Death Dis.

[22] Xu Y., Jagannath C., Liu X.-D., Sharafkhaneh A., Kolodziejska K.E., Eissa N.T. (2007). Toll-like receptor 4 is a sensor for autophagy associated with innate immunity. Immunity.

[23] Dunlop E.A., Tee A.R. (2014). mTOR and autophagy: a dynamic relationship governed by nutrients and energy. Semin. Cell Dev. Biol.

[24] Comb W.C., Hutti J.E., Cogswell P., Cantley L.C., Baldwin A.S. (2012). p85α SH2 domain phosphorylation by IKK promotes feedback inhibition of PI3K and Akt in response to cellular starvation. Mol. Cell.

[25] Yang F., Tang E., Guan K., Wang C.Y. (2003). IKKβ plays an essential role in the phosphorylation of RelA/p65 on serine 536 induced by lipopolysaccharide. J. Immunol.

[26] Dauphinee S.M., Karsan A. (2006). Lipopolysaccharide signaling in endothelial cells. Lab. Invest.

[27] Dan H.C., Cooper M.J., Cogswell P.C., Duncan J.A., Ting J.P.Y., Baldwin A.S. (2008). Akt-dependent regulation of NF-κB is controlled by mTOR and Raptor in association with IKK. Genes Dev.

[28] Burke J.R., Pattoli M.A., Gregor K.R., Brassil P.J., MacMaster J.F., McIntyre K.W., Yang X., Iotzova V.S., Clarke W., Strnad J., Qiu Y., Zusi F.C. (2003). BMS-345541 is a highly selective inhibitor of IκB kinase that binds at an allosteric site of the enzyme and blocks NF-κB–dependent transcription in mice. J. Biol. Chem.

[29] Hutti J.E., Turk B.E., Asara J.M., Ma A., Cantley L.C., Abbott D.W. (2007). IκB kinase β phosphorylates the K63 deubiquitinase A20 to cause feedback inhibition of the NF-κB pathway. Mol. Cell Biol.

[30] Lizcano J.M., Göransson O., Toth R., Deak M., Morrice N.A., Boudeau J., Hawley S.A., Udd L., Mäkelä T.P., Hardie D.G., Alessi D.R. (2004). LKB1 is a master kinase that activates 13 kinases of the AMPK subfamily, including MARK/PAR-1. EMBO J.

[31] Hawley S.A., Davison M., Woods A., Davies S.P., Beri R.K., Carling D., Hardie D.G. (1996). Characterization of the AMP-activated protein kinase kinase from rat liver and identification of threonine 172 as the major site at which it phosphorylates AMP-activated protein kinase. J. Biol. Chem.

[32] Menon M.B., Dhamija S. (2018). Beclin 1 phosphorylation: at the center of autophagy regulation. Front. Cell Dev. Biol.

[33] Kim J., Kim Y.C., Fang C., Russell R.C., Kim J.H., Fan W., Liu R., Zhong Q., Guan K.L. (2013). Differential regulation of distinct Vps34 complexes by AMPK in nutrient stress and autophagy. Cell.

[34] Schaaf M.B., Keulers T.G., Vooijs M.A., Rouschop K.M. (2016). LC3/GABARAP family proteins: autophagy-(un)related functions. FASEB J.

[35] Kaizuka T., Morishita H., Hama Y., Tsukamoto S., Matsui T., Toyota Y., Kodama A., Ishihara T., Mizushima T., Mizushima N. (2016). An autophagic flux probe that releases an internal control. Mol. Cell.

[36] Ni H.-M., Bockus A., Wozniak A.L., Jones K., Weinman S., Yin X.-M., Ding W.-X. (2011). Dissecting the dynamic turnover of GFP-LC3 in the autolysosome. Autophagy.

[37] Nussenzweig S.C., Verma S., Finkel T. (2015). The role of autophagy in vascular biology. Circ. Res.

[38] Guo F., Li X., Peng J., Tang Y., Yang Q., Liu L., Wang Z., Jiang Z., Xiao M., Ni C., Chen R., Wei D., Wang G.X. (2014). Autophagy regulates vascular endothelial cell eNOS and ET-1 expression induced by laminar shear stress in an ex vivo perfused system. Ann. Biomed. Eng.

[39] Amaravadi R.K., Thompson C.B. (2007). The roles of therapy-induced autophagy and necrosis in cancer treatment. Clin. Cancer Res.

[40] Degtyarev M., De Mazière A., Orr C., Lin J., Lee B.B., Tien J.Y., Prior W.W., van Dijk S., Wu H., Gray D.C., Davis D.P., Stern H.M., Murray L.J., Hoeflich K.P., Klumperman J. (2008). Akt inhibition promotes autophagy and sensitizes PTEN-null tumors to lysosomotropic agents. J. Cell Biol.

[41] Rahimi N., Dayanir V., Lashkari K. (2000). Receptor chimeras indicate that the vascular endothelial growth factor receptor-1 (VEGFR-1) modulates mitogenic activity of VEGFR-2 in endothelial cells. J. Biol. Chem.

