# Supplementary material for: Cell adhesion molecule IGPR-1 activates AMPK connecting cell adhesion to autophagy
Source: J Biol Chem. 2021 Jan 13;295(49):16691–9. doi: 10.1074/jbc.RA120.014790 (PMC7864065; doi:10.1074/jbc.RA120.014790)

S. Figure 1. **IKKβ selectively phosphorylates IGPR-1 at Ser220**. Cell lysates from HEK-293 cells expressing either wild type IGPR-1, A220-IGPR-1 or D220-IGPR-1 were subjected to immunoprecipitation. The immunoprecipitated proteins were subjected to calf intestinal alkaline phosphatase (CIP) treatment which removes phosphorylation. The removal of phosphorylation on IGPR-1 was confirmed by Western blot analysis using phospho-Ser220 specific antibody (**A**). The dephosphorylated proteins were subjected to an *in vitro* kinase assay using a recombinant IKKβ (**B**). The result showed that IKKβ selectively phosphorylates IGPR-1 at Ser220.

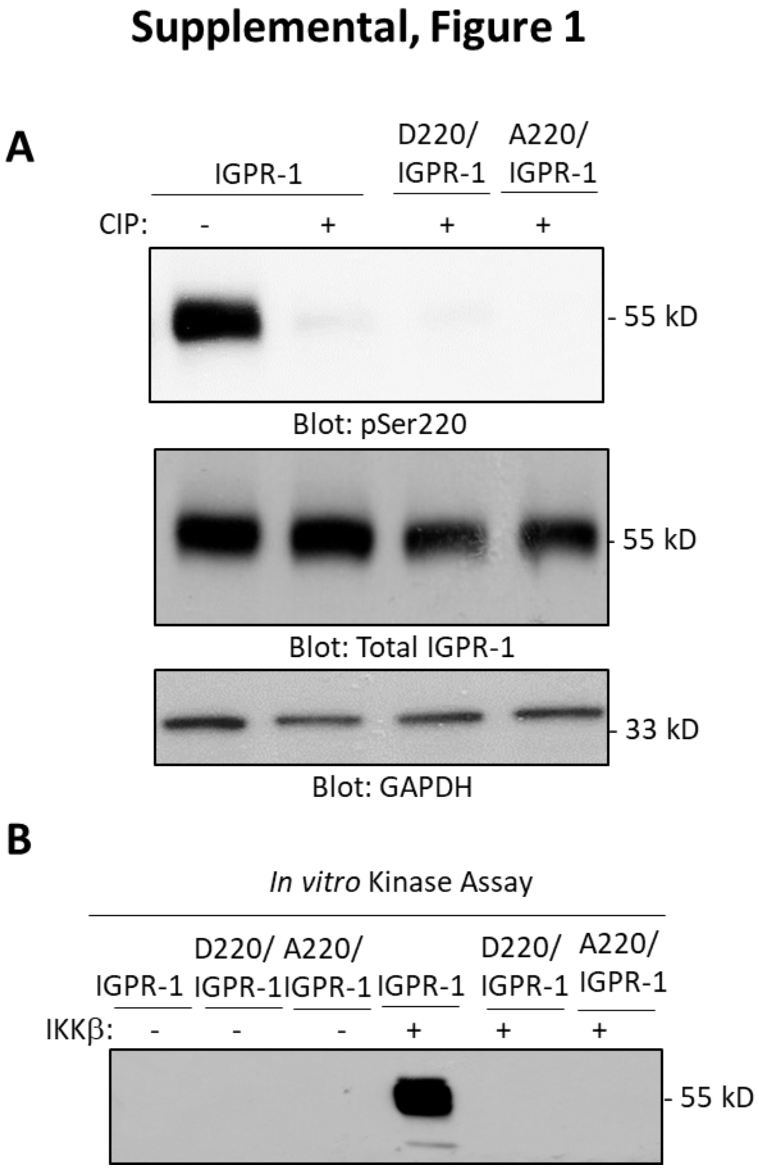

Supplement: Supplementary file 1 [file mmc1.pdf]
